# The Effects of Yoga on the Mental Health of Individuals With Autoimmune Disorders: A Scoping Review

**DOI:** 10.7759/cureus.77669

**Published:** 2025-01-19

**Authors:** Stephanie Nagy, Kelsey Tague, Angie Ossorio, Niyati Patel, Ryan Callahan, Elizabeth Jose, Mary Tran, Ashley Mejia, Megan Centrella, Marissa N McPhail, Jacqueline Junco, Marc M Kesselman

**Affiliations:** 1 Rheumatology, Nova Southeastern University Dr. Kiran C. Patel College of Osteopathic Medicine, Davie, USA; 2 Internal Medicine, Nova Southeastern University Dr. Kiran C. Patel College of Osteopathic Medicine, Davie, USA; 3 Osteopathic Medicine, Nova Southeastern University Dr. Kiran C. Patel College of Osteopathic Medicine, Davie, USA; 4 Neuroimmune Medicine, Nova Southeastern University Dr. Kiran C. Patel College of Osteopathic Medicine, Davie, USA

**Keywords:** autoimmune disorder, complementary and integrative health, irritable bowel disease, mental health, multiple sclerosis, rheumatoid arthritis, yoga therapy

## Abstract

Yoga has been explored as a health modality to maintain mental and physical health and as a complementary therapy for managing various medical conditions. It has been most recently researched for its ability to decrease inflammatory markers responsible for various ailments. This opens the door for its potential role as an adjunct therapy in inflammatory-led dysfunctions such as autoimmune disorders. Although there has been a robust amount of data on yoga and autoimmune conditions, previous reviews have mostly been limited to the physical improvements that patients experience rather than the mental health changes. This scoping review aims to address the role of yoga as an effective therapy choice in treatment and management options for the mental health symptoms associated with autoimmune disorders. The initial search revealed 211 relevant studies, but following an extensive review, 11 articles were included in the final analysis. Yoga interventions ranged from six weeks to up to six months and included Iyengar yoga, Hatha yoga, and generalized yoga practices that did not fit into a specific subtype. Eight articles analyzed patients with multiple sclerosis, two focused on rheumatoid arthritis and one assessed irritable bowel disease. Between the 11 studies included in this review, the key markers analyzed were stress level, anxiety, sleep, self-efficacy, depression, and emotional fatigue. Significant improvement was found in all these subtypes. Yoga is a viable, non-pharmaceutical treatment for both the physical and mental health components of patients with autoimmune disorders.

## Introduction and background

Autoimmune disorders affect around 1 out of 10 individuals, with the highest prevalence being among women. These disorders arise from a combination of multiple factors, including genetics, environmental, infections, hormonal, and high stress levels, all of which increase the risk of autoimmune disorders [1]. When an already weakened immune system encounters these risk factors, it makes the body susceptible to an attack on itself.

The immune system has three primary functions: to identify and protect the body from foreign matters, to recognize and keep a record of past infections, and to prevent an attack on the body’s own cells. In this intricate system, multiple key players must interact in a regulated manner to work against a common enemy [2]. In most individuals, a state of tolerance and a sufficient T-cell response prevents the body from attacking itself. When the ability to recognize your own cells is lost, an increase in autoreactive T cells comprises the immune system's ability to differentiate between healthy and harmful cells. An increase in autoantibodies against the host weakens the immune system, causing a widespread immune response affecting multiple healthy organs and tissues. Thus, the immune system mistakenly attacks healthy cells, leading to the development of autoimmune disorders [3].

Conventionally, autoimmune conditions are treated with pharmaceuticals, specifically immunosuppressive medications. The treatment for each autoimmune condition does vary; however, a staple in many of the treatments is corticosteroids. They are often the first line of treatment due to their anti-inflammatory effects. However, the lowest dose is often recommended due to the severe side effects that follow with long-term use and higher dosages, including opportunistic infections, psychosis, weight gain, hypertension, pancreatitis, hyperlipidemia, and hyperglycemia [4]. Immunosuppressive agents are often used with corticosteroids, including azathioprine, mycophenolate mofetil, and calcineurin inhibitors. They are better tolerated than corticosteroids but also have their own set of adverse effects, including infections, nausea, vomiting, anemia, leukopenia, thrombocytopenia, tremors, malignancies, and hypertension [5-7]. Newer drugs that have come onto the market more recently include biological agents, including TNF-alpha inhibitors (infliximab, adalimumab, and etanercept), interleukin (IL) inhibitors targeting IL-1,6, 12, 17 and 23, B cell depleting agents, and chimeric antigen receptor T cell (CAR-T) therapy. Biologic therapies have been found to have the least severe side effects, with infection being the most common adverse effect [8].

As a result of complications and mainstream media, complementary and alternative medicine therapies have risen in popularity in the United States in recent decades. Patients have sought a variety of these therapies over the years, including herbal medicine and acupuncture. Herbal medicine, for instance, dates back to ancient times in several cultures. People heavily depended on roots and plants due to the versatility of their use. Soon enough, people began to observe the potential therapeutic effect of these plants. The oldest evidence of the use of medicinal plants was documented on a Sumerian clay slab about five thousand years ago [9]. Traditional Chinese Medicine incorporates herbal medicine along with several other alternative therapies, such as acupuncture and different forms of exercise, including Tai Chi and Qigong [10]. People commonly seek the more natural side of medicine in pursuit of wellness-oriented care, with a holistic approach, as well as to avoid the adverse effects of traditional medicine. Several of these techniques involve not only a physical component of healing but a spiritual component as well, which appeals to people who try to improve the health of their mind, body, and soul [11]. Many people have also turned to acupuncture as another method. It is a form of traditional Chinese medicine that involves placing needles on specific points of the body. In patients with autoimmune conditions, it has been found to suppress the immune system through modulating cells within the adaptive and innate immune systems [12]. Novel studies have found that acupuncture may reduce pain and fatigue in patients with autoimmune conditions [13]. Now, more so than ever, the increased access to information enables people to be more informed of alternative treatments and be part of communities that support similar health philosophies.

Yoga is one of the most practiced complementary therapies, approximately 35.2 million Americans practiced yoga in 2017 [14]. The trend has been increasing in recent decades and is expected to continue to rise. It is an ancient practice that originates from India and was created to unite the body with the mind to promote health [15,16]. Originally, yoga was intended to be used as a tool for holistic palliatives for healing. It has since been mainstreamed in other parts of the world as a form of physical exercise consisting of breathwork and meditation [15]. Yoga has emerged as a new tool to improve and maintain mental and physical health [17-21]. Several different types of yoga offer a unique approach to achieving the connection between mind, body, and one’s breathing. One of the most popular techniques practiced in the world is Hatha yoga, a general technique of any physical yoga that involves different seated and standing positions with focus on strength, flexibility, and relaxation. Another popular technique is hot yoga, which offers the additional challenge of performing yoga in a heated environment. The purpose of the heat is to allow the body to stretch beyond its usual flexibility, as well as detoxification of the body through sweating. Other techniques of yoga that are commonly practiced are Vinyasa yoga, Ashtanga yoga, restorative yoga, Iyengar yoga, and many other existing styles [22].

The physical effects of yoga are well known, and the mental benefits are now being further investigated. In seniors, yoga was found to reduce stress and fatigue and improve quality of life [20,23-25]. Beyond specific populations, yoga has played an influential role in the well-being of the public. Research shows that yoga can improve emotional functioning in those healthy or ill and their quality of life [26,27]. Practicing yoga regularly can enhance mood and promote happiness through mechanisms triggering neurotransmitters that modulate anxiety [28]. Yoga also allows for the opportunity to practice mindfulness, allowing individuals to focus on the present while also reducing anxiety. Yoga’s emotional and psychological benefits not only allow for therapeutic use for specific populations but also are widely available for the general population to utilize.

Autoimmune diseases are typically accompanied by chronic inflammation, due to dysregulation of the body’s immune response or defective self-tolerance. Several disease markers are often seen in autoimmune diseases, such as IL-6, IL-1β, and tumor necrosis factor-𝛼 (TNF-𝛼), with IL-6 being one of the key markers [29]. TNF-𝛼 is a powerful pro-inflammatory cytokine that enhances T-cell differentiation and survival at sites of inflammation [30]. IL-6 is produced in the early stages of inflammation and induces the synthesis of acute-phase proteins such as serum amyloid A, C-reactive protein (CRP), TNF-𝛼, fibrinogen, and hepcidin in the liver. High amounts of serum amyloid A that remains long enough in the bloodstream can lead to the buildup of amyloid in various organs, which is a common complication in autoimmune disorders [31]. CRP is a key acute-phase reactant involved in the inflammatory pathway, rapidly increasing in response to pro-inflammatory cytokines like IL-6, IL-1β, and TNF-α. It facilitates immune defense by promoting complement activation, enhancing phagocytosis through opsonization, and modulating cytokine production [32]. The interaction of CRP with leukocytes and endothelial cells and the subsequent release of more pro-inflammatory cytokines has been well established [33]. Similar to CRP, the erythrocyte sedimentation rate (ESR) is almost a significant marker for inflammation and can indicate flare-ups [34].

The inflammatory markers can produce a toxic environment for cells leading to inflammation and pathology in the tissues involved [35]. These pathologies can include the skin, musculoskeletal system, and neurological system [36]. The limitations on movement and overall tasks of daily living consequently contribute to a lower quality of life and even depression in these individuals [36,37]. Disease markers are common indications used to determine the severity, progression, and remission of a disease. Monitoring inflammatory markers is a way of tracking disease activity in these various autoimmune disorders [35,38]. Notably, excessive secretion of pro-inflammatory markers versus reduced secretion of anti-inflammatory markers has been attributed to the disease process of autoimmune conditions [38]. The pro-inflammatory markers include IL-6, while the anti-inflammatory markers include IL-17 [38]. It has been found that those who practice yoga regularly had decreased levels of inflammatory markers CRP, ESR, and IL-6 than those new to yoga [39-42]. This makes it plausible to suggest that the regular practice of yoga decreases these inflammatory markers and thus indicates it as a treatment option for autoimmune pathologies involving these markers.

It has been proposed that yoga may help reduce pain levels and overall quality of life in individuals with autoimmune disorders [25]. Gautam and colleagues explored how the practice of yoga significantly decreases disease activity, pain due to chronic disease, swelling, affiliated disability, and stress. Yoga resists the stress response, aids in endocrine hormone homeostasis, and preserves the body's joint mobility and overall flexibility. They found that the scores representing disease activity, pain acuity, and disability showed a statistically significant enhancement in patients with rheumatoid arthritis who participated in eight weeks of yoga-based lifestyle interventions. Their findings suggest that yoga has the potential to be practiced as an additive symptomatic treatment option in patients with rheumatoid arthritis [17]. Ahmadi and colleagues analyzed the physical symptoms and limitations that often hinder patients with multiple sclerosis, such as mobility, gait disturbances, difficulty walking, and fatigue [41]. Patients with multiple sclerosis who underwent three one-hour sessions for eight weeks showed improvement in balance, walking endurance, and fatigue symptoms, along with decreased depression and anxiety. Practicing yoga can improve overall ambulatory functioning, fatigue, and mood in patients with multiple sclerosis by encouraging muscle relaxation, improving cardiovascular and respiratory proficiency, and reducing muscle spasticity [43]. Understanding the impact of yoga on the physical symptoms of autoimmune conditions, however, as chronic conditions play a significant role in the mental health of individuals, it is crucial to investigate this aspect as patients with autoimmune conditions were found to develop different forms of mental health conditions due to chronic pain, stress and lack of sleep.

Overall, there is strong enough evidence for further exploration of the potential role of yoga as an adjunctive therapy for its impact on mental health due to the toll autoimmune disorders cause physically as well as mentally. As a result, in this scoping review, we aim to identify the current findings within this topic to better understand yoga’s effectiveness in treating the mental health of patients with an autoimmune disorder.

## Review

Methods

Search Strategy

The literature search was conducted in 2023 and composed of articles published from January 2000 to November 2023. The inclusion criteria were as follows: English language and free full-text articles, articles including participants aged 18 to 64 years, and the inclusion of the keywords within the title and abstract. The research includes primary and secondary sources, mainly academic publications identified via professional databases including EMBASE, Ovid MEDLINE, Web of Science, and CINAHL. The keywords selected for the literature search were as follows: (yoga) AND ((autoimmune disorders) OR (rheumatoid arthritis) OR (multiple sclerosis) OR (lupus*) OR (Systemic lupus erythematosus) OR (type I diabetes mellitus) OR (celiac*) OR (Hashimoto) OR (Hashimoto thyroiditis) OR (graves*) OR (inflammatory bowel disease)). Relevance was evaluated using a hierarchical approach, which evaluated the title, abstract, and then the full manuscript. 

The studies included were considered eligible if they investigated the selected autoimmune disorders and yoga. Eligible study designs include randomized control trials, case-control studies, case studies, and prospective/retrospective studies. Inclusion criteria included free full-text versions, articles in the English language, adults aged 18 to 64 and the inclusion of keywords within the title and abstract. Exclusion criteria included study designs which were secondary analysis-based studies and literature reviews, inaccessibility of the full-text version, and duplicate studies.

Study Selection Process

**Figure 1:**
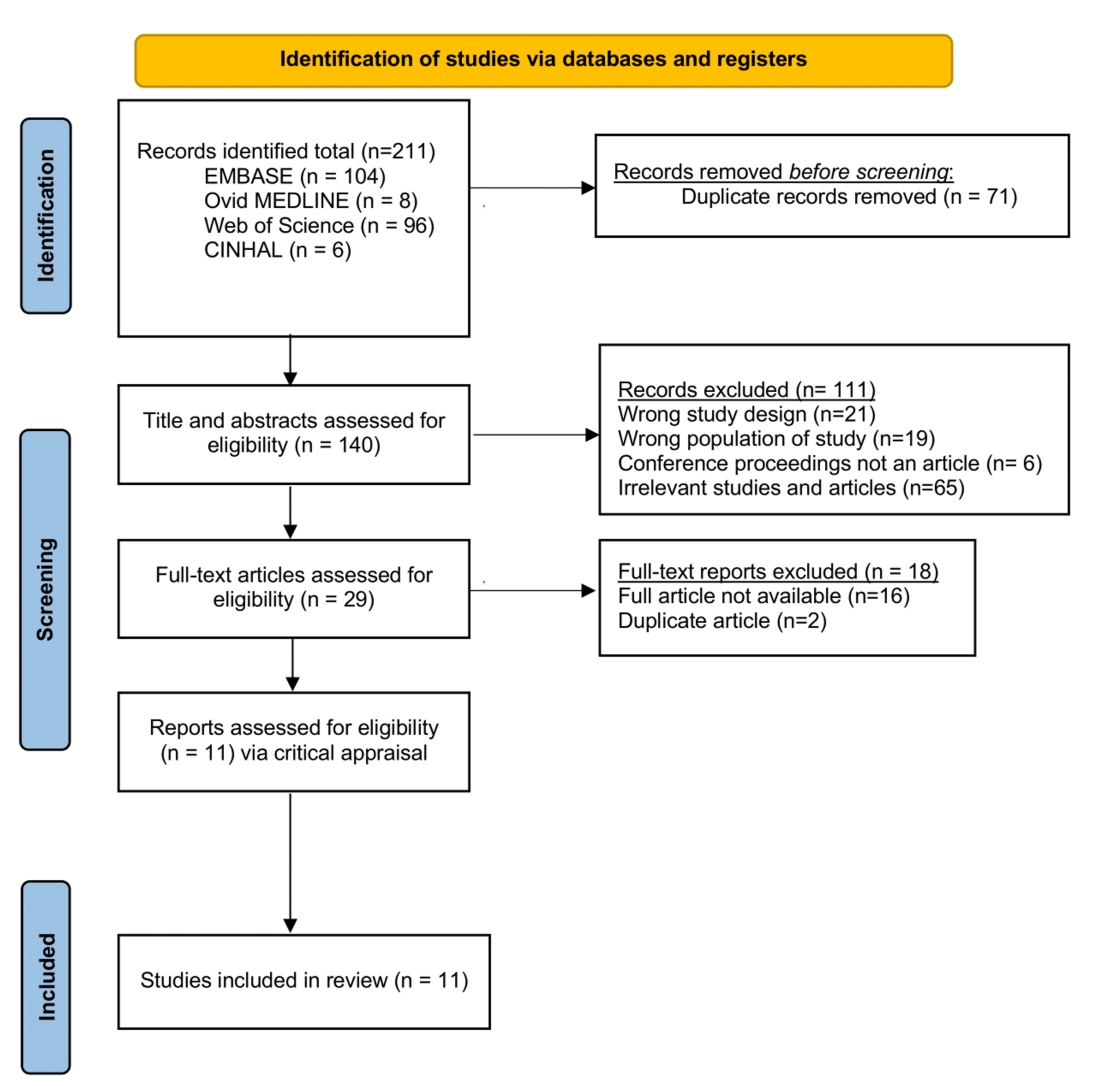
PRISMA Article Selection Diagram PRISMA: Preferred Reporting Items for Systematic reviews and Meta-Analyses

The initial search revealed 211 relevant citations. Initially, 71 duplicate citations were removed and the remaining 140 were assessed for eligibility. Four authors analyzed the abstracts and determined the eligible articles for further analysis (n=140 articles), of which n=113 were excluded for wrong study design, wrong population being studied, and being published as conference proceedings. This scoping review only considered experimental studies including randomized controlled trials and non-randomized controlled trials. Reviews, meta-analyses, abstracts, conference presentations, commentaries, case reports, and letters to the editors were excluded. Two authors then proceeded to review the remaining full-text articles, for proper inclusion criteria. Any disagreement between the reviewers prompted the article review by a third-party reviewer, an agreement was reached when the three reviewers arrived at a decision through discussion and mutual evaluation of the studies. After the second-tier review, 11 articles were included for analysis. The screening and selection process was depicted using the PRISMA flowchart in Figure 1 [44]. The Joanna Briggs Institute Critical Appraisal Tool was used to analyze the reliability and efficacy of the 11 studies, and all 11 were seen to be of satisfactory quality and validity for the review (Table 1).

**Table 1:** Joanna Briggs Institute Critical Appraisal of the Included Articles

	Is the review question clearly and explicitly stated?	Were the inclusion criteria appropriate for the review question?	Was the search strategy appropriate?	Were the sources and resources used to search for studies adequate?	Were the criteria for appraising studies appropriate?	Was critical appraisal conducted by two or more reviewers independently?	Were there methods to minimize errors in data extraction?	Were the methods used to combine studies appropriate?	Was the likelihood of publication bias assessed?	Were recommendations for policy and/or practice supported by the reported data?	Were the specific directives for new research appropriate?	Score	Quality
Ahmadi et al. [41]	Yes	Yes	Yes	Yes	Yes	Yes	Yes	Yes	Yes	Yes	Yes	11	High
Cartwright et al. [45]	Yes	Yes	Yes	Yes	Yes	Yes	Yes	Yes	Yes	Yes	Yes	11	High
de Oliveira et al. [46]	Yes	Yes	Yes	Yes	Yes	Yes	Yes	Yes	Yes	Yes	Yes	11	High
Donnelly et al. [47]	Yes	Yes	Yes	Yes	Yes	Yes	Yes	Yes	Yes	Yes	Yes	11	High
Evans et al. [48]	Yes	Yes	Yes	Yes	Yes	Yes	Yes	Yes	Yes	Yes	Yes	11	High
Hasanpour-Dehkordi et al. [49]	Yes	Yes	Yes	Yes	Yes	Yes	Yes	Yes	Yes	Yes	Yes	11	High
Kahraman et al. [50]	Yes	Yes	Yes	Yes	Yes	Yes	Yes	Yes	Yes	Yes	Yes	11	High
Kaur et al. [51]	Yes	Yes	Yes	Yes	Yes	Yes	Yes	Yes	Yes	Yes	Yes	11	High
Najafi et al. [52]	Yes	Yes	Yes	Yes	Yes	Yes	Yes	Yes	Yes	Yes	Yes	11	High
Pan et al. [53]	Yes	Yes	Yes	Yes	Yes	Yes	Yes	Yes	Yes	Yes	Yes	11	High
Razazian et al. [54]	Yes	Yes	Yes	Yes	Yes	Yes	Yes	Yes	Yes	Yes	Yes	11	High

Results

Eight hundred and seventy-six patients were analyzed in the studies. Seven of the studies indicated the sex distribution of the patients with four of them not specifying. The majority of the patients identified were female, which is an expected finding within autoimmune conditions. Of the 11 studies included in this review, five assessed the impact of yoga on stress levels in patients with autoimmune conditions [45,46,49,52,54], six assessed the impact of yoga on anxiety [41,47,48,49,51,52], three assessed the impact of yoga on self-efficacy [47-49], six assessed the impact of yoga on depression [45,48,50,52,54], and seven of the studies assessed the impact of yoga on emotional fatigue in autoimmune patients [41,47-50,53,54]. Most of the studies looked at patients with multiple sclerosis (MS) [41,46,47, 49,50,53,54], with two focusing on patients with rheumatoid arthritis (RA) [45,48], and one assessed irritable bowel disease [51]. Three hundred and sixty-nine participants with autoimmune conditions were studied across all the articles. Of this 369, 275 were women, 34 men, and 60 of unknown sex. Three hundred and twenty-four of the participants had a diagnosis of MS, 36 had RA, and nine had irritable bowel disease (6 with Crohn’s and 3 with ulcerative colitis). Yoga interventions ranged from six weeks to up to six months and included Iyengar yoga, Hatha yoga, and generalized yoga practices that did not fit into a specific subtype. Typically, these interventions consisted of 1-3 sessions per week. Table 2 analyzes the studies that were examined for this scoping review. 

**Table 2:** Analysis of Studies

Authors	Purpose	Study Design	Study Population	Sex of Patients (F= Female, M=Male)	Mean Age in Years	Methods	Limitations	Key Findings
Ahmadi et al. [41]	To investigate the impact of aerobics and yoga on ambulatory function, fatigue, and mood of multiple sclerosis patients.	Randomized controlled trial	Multiple sclerosis patients with the Expanded Disability Status Scale score between 1 and 4.	31 F	36.75	Randomized trial split into three groups: 1) treadmill training, 2) yoga, and 3) control. Yoga and aerobic training three times a week for eight weeks.	Study only included women	Results showed that pre- and post-interventions produced significant improvements in the Fatigue Severity Scale score, Beck Depression Inventory score and Beck Anxiety Inventory score in the treadmill training group and yoga group. Yoga groups showed a larger decrease in Beck Anxiety Inventory scores.
Cartwright et al. [45]	To explore patients’ experiences of individualized yoga therapy intervention for rheumatoid arthritis.	Non-randomized pilot study	Adults 18 or older with rheumatoid arthritis for at least three months	9 F, 1 M	53.6	Single-group pre-, post (four months) and follow-up (12 months) design, with in-depth interviews conducted post intervention. 10 one hour 1-to-1 consultations over a 16-week period followed by two group review sessions (at five and nine months).	Small sample size and no control group used.	Yoga therapy has potential as an adjunct therapy to improve rheumatoid arthritis symptoms, increase self-care behaviors and manage stress.
Oliveira et al. [46]	To investigate the impact of yoga on postural balance and how that affects daily activities of those with multiple sclerosis.	Randomized controlled study	People who are yoga naive and have multiple sclerosis.	560 –Did not specify sex	Did not indicate the mean age, but all participants included in the study were above 65	Yoga group practiced postures, breathing exercises, meditation, and relaxation in weekly 60-min classes for a six-month period.	Many variables, including attention from the yoga instructor, could have interfered with the results. Also, there was a small sample size.	There was an improvement in the Berg Balance Scale score only in the Yoga group. There was increased quality of self-reported postural balance, and decreased influence of postural imbalance on daily activities.
Donnelly et al. [47]	To investigate the effectiveness of the LoveYourBrain yoga program on those with multiple sclerosis and how it could improve their health outcomes.	Non-randomized controlled study	Adults fluent in English with an Expanded Disability Status Scale score of ≤ 6. Also had to be medically stable for eight weeks prior to the experiment.	13 F, 2 M	Mean age not provided only stratification of age range 24-34 years = 2 35-44 years= 2 44-54 years= 6 55+=4	Non-randomized, 2x2 crossover pilot trial compared the LoveYourBrain Yoga program to a control condition with a three-week washout period among individuals with multiple sclerosis. Six-week, manualized, group-based yoga with psychoeducation program that includes 10 min of breathing exercises, 45 min of gentle yoga, 15 min of meditation, and 20 min of group discussion with psychoeducation.	Non-randomized and small sample size. Lack of heterogeneity among the participants in terms of gender, race, and ethnicity.	Significant improvements in fatigue, positive affect and wellbeing, and anxiety were found after LoveYourBrain.
Evans et al. [48]	To explore the impact of Iyengar yoga on the quality of life of those with rheumatoid arthritis.	Randomized controlled study	Female adult patients between 16 and 35 years old with rheumatoid arthritis for at least six months.	35 – Did not specify the sex	Did not indicate the mean age	The Iyengar yoga program consisted of six weeks of 1.5-hour classes held twice per week. Health-related quality of life of young adults with rheumatoid arthritis compared with a usual-care waitlist control.	N/A	Iyengar yoga intervention is a feasible and safe adjunctive treatment for young people with rheumatoid arthritis, leading to health-related quality of life, pain disability, fatigue, and mood benefits.
Hasanpour-Dehkordi et al. [49]	To investigate the effect of yoga on patients with multiple sclerosis and evaluate changes in physiological indices, anxiety, and social function in Iran.	Single-blind controlled trial	People with multiple sclerosis	90 – Did not specify the sex	Did not indicate mean age	Yoga exercise sessions were three 60- to 70-minute sessions per week for 12 weeks. The exercise started with light music. There was an emphasis on breathing for concentration and relaxation during the session. Each session ended with a 10-minute-deep relaxation.	N/A	Yoga is likely to increase self-efficacy of multiple sclerosis patients through enhancing physical activity, increasing the strength of lower limbs and balance, and decreasing fatigue and pain, and finally to promote social functioning and to relieve stress and anxiety.
Kahraman et al. [50]	To investigate the effectiveness of a six-month yoga program in improving the physical and psychosocial symptoms of participants with multiple sclerosis and their family members.	Uncontrolled clinical trial	People with multiple sclerosis	27 - Did not specify the sex	Did not indicate mean age	Once a week for six months, participants had yoga training (for at least one hour). Assessors measured at baseline and immediately after the end of the yoga program.	Absence of a control group. The study has a small sample size and low participation rate. It was difficult to generalize results to the whole population since most people with multiple sclerosis are female. Information about personal reasons for dropping out of the study were not collected in a questionnaire.	Participants with multiple sclerosis showed significant improvement in mental dimension of health-related quality of life, walking speed, fatigue and depression after the yoga program. There was no significant change in participants with multiple sclerosis in self-reported walking impact, balance, pain, physical dimension of health-related quality of life, and kinesiophobia levels.
Kaur et al. [51]	To examine the acceptability, implementation, and effectiveness of an integrated eight-week yoga program and aromatherapy massage in patients with inflammatory bowel disease.	Single-center pre- and post- intervention pilot study	Patients with inflammatory bowel disease above 18 years old	8 F, 1 M	52.1	Patients participated in weekly 30-minute yoga sessions for eight weeks. Participants were asked to complete the same practices daily at home. Patients' anxiety, mental and physical health, perceived stress, sleep quality,and depression levels were measured using surveys and questionnaires.	The study may not be able to be used to determine intervention effectiveness due to the small sample size. Study has a lack of generalizability and absence of a control group.	Mean sleep scores decreased suggesting some sleep improvement. At baseline, participants reported mild anxiety symptoms, mild to moderate depressive symptoms, moderate levels of perceived stress, and poor sleep quality. After the intervention, participants reported decreased depression, and anxiety scores, and increased mental component scores and physical component scores.
Najafi et al. [52]	To investigate the effects of eight weeks of tele-yoga and tele-pilates on the serum levels of prolactin and cortisol and selected physical and psychological factors.	Single blinded randomized control trial	Patients relapsing multiple sclerosis.	15 F	37.4	Patients were randomized to either tele-pilates, tele-yoga or control groups. Patients underwent three sessions a week for eight weeks. Each session included three phases: warming up (10–15 min), main poses (30–40 min), and cooldown (10–15 min). Serum blood samples and validated questionnaires were collected before and after interventions. Outcomes measured included prolactin level, cortisol level, depression score, physical activity levels, quality of life, speed of walking.	The study may not be generalizable to other types of multiple sclerosis since it only assessed tele-exercise for relapsing multiple sclerosis.	A significant decrease in depression scores were seen after tele-exercising. Quality of Life improved following tele-yoga and tele-Pilates in female patients with multiple sclerosis. Mental health comparison showed more changes in tele-yoga exercise than in tele-Pilates training in females with multiple sclerosis.
Pan et al. [53]	To assess the effects of Baduanjin exercise and yoga on balance ability, posture control, fatigue and depression in patients with multiple sclerosis. Secondary goal is to determine if Baduanjin is better than yoga at controlling emotions, relieving depression and improving balance ability.	Prospective, randomized, controlled study	Multiple sclerosis patients with the Expanded Disability Status Scale score less than 5.	21 F, 9 M	40.93	The patients were randomly organized into yoga exercises, Baduanjin exercises, or a control group for 24 consecutive weeks. Patients' depression, fatigue and disability status were measured. The yoga group took sessions once a day for about 60 min for 24 consecutive weeks under the supervision of a certified yoga instructor.	This study was a single-center study and was not a blind trial.	In the yoga group, the number of patients who had depressive symptoms decreased after intervention. In the yoga group, patients in the pre-exercise and in the post-exercise period who were classified as exhibiting fatigue had significantly decreased fatigue scores after the intervention.
Razazian et al. [54]	Assess if exercise intervention programs have a positive impact on fatigue, depression, and paresthesia in patients with multiple sclerosis.	Randomized blinded clinical trial	Patients with multiple sclerosis.	54 F	33.94	Patients were randomized to either yoga, non-exercise, or aquatic exercise groups. Yoga sessions took place three times a week for about 60 min for eight consecutive weeks under the supervision of a certified yoga instructor (Hatha yoga). Patients were assessed for fatigue, depression, and paresthesia.	The study had a small sample size and exclusively assessed female patients. No biomarkers were assessed, no sleep was assessed, no data was collected for muscle strength, cardiovascular changes, or gait. Symptoms of depression were only assessed by self-ratings.	Fatigue, depression, and paresthesia decreased from the beginning of the study to the end of the study eight weeks later in the yoga group in comparison to the control group in all aspects. Yoga and aquatic exercise had equal reductions in depression and paresthesia, but yoga had a greater reduction in fatigue when compared to the aquatic exercise group.

The common themes analyzed by the articles included stress, anxiety, sleep, depression, self-efficacy, and emotional fatigue.

Impact on stress

Several studies showed that yoga helped improve stress associated with a patient’s autoimmune disorder [45,46,50,52,54]. Some studies showed that participants associated improved stress with enhanced quality of life and better sleep [45,49,52]. Patients who did not participate in yoga were found to respond to stress with more anger and those who completed yoga therapy were generally less stressed as well [49]. Cartwright et al. reported that participants with RA attributed improvements in stress to yoga’s ability to reframe thoughts, leading to a reduced reactivity stress response [45]. On the other hand, one study by Kaur et al. found no significant change in reported stress in yoga participants with inflammatory bowel disease [51].

Impact on anxiety

Yoga significantly decreased anxiety across multiple studies [41,47,48,49,51,52]. Evans et al. used a numeric rating scale of 0-10 to assess the participants’ levels of anxiety every week; findings indicated improved anxiety levels that continued to be maintained throughout follow-up visits [48]. Using the General Anxiety Disorder-7 assessment, a significant reduction from a score of 5.4 to 4.2 was noted [51]. Furthermore, the Beck Anxiety Inventory assessment found a significant score reduction of 48.56% from 12.45 to 6.45 (p=0.001) in reported anxiety in MS patients who completed the yoga program [29]. These studies suggest that quality of life may be improved because of reduced anxiety after yoga [49,52].

Impact on sleep

Most studies did not report significant improvements in sleep outcomes among those with autoimmune disorders [47,48,51]. Evans et al. indicated insignificant scores in changes to sleep difficulty [48]. Furthermore, there were insignificant changes in sleep disturbance in those with MS and inflammatory bowel disease [48,51]. Additionally, one of the five participants partaking in yoga reported a decrease in sleep quality [51]. However, Cartwright et al. found that participants attributed improved sleep due to perceived reduced stress and physical pain following yoga [45].

Impact on depression

Six of the studies evaluated the effects of yoga on depression in patients with autoimmune conditions [41,45,48,50,52,53]. Using the Zung Self-Rating Depression Scale, following a 24-week yoga regime, the number of patients with depression symptomology reduced from 20 to 7 [52]. Also, with the use of the Beck Depression Inventory (BDI) assessment, there was a significant decrease in depression with the use of tele-yoga by 8 fold, also a 36.11% reduction in BDI scores and a significant reduction in BDI scores from 10 to 5 [41,50,52]. When scoring depression feelings on a scale from 0-10 (0 being no depression and 10 being the worst possible depression), Evans et al. noted a mild decrease from a self-reported score of 1.8 to 1.0 at the end of the intervention [48].

Impact on self-efficacy

Improvement in self-efficacy after engaging in yoga was reported to improve in three of the articles [47-49]. A study on people with MS reported significant improvement in the self-efficacy of participants through yoga interventions with the use of the quality of life questionnaire SF-36 [49]. The Arthritis Self-Efficacy Scale (ASES) was also used and reported a significant reduction in the ASES pain score from 12.96 baseline to 15.68 after six weeks [48]. Self-efficacy was also assessed in another study using the 11-item Liverpool Self-Efficacy scale; improvements were noted. However, they were non-significant [47].

Impact on emotional fatigue

Significant reductions in emotional fatigue were reported in most studies [41,47-50,53,54]. Two articles reported using the Fatigue Severity Scale finding significant decreases in fatigue in participants who practiced yoga from 33.3% to 58.3% [52,53]. One article used the PROMIS-Fatigue MS scale also reporting reductions in fatigue and improved quality of life [47].

Evans et al. used the Functional Assessment of Chronic Illness Therapy Fatigue Subscale (FACIT-Fatigue) to assess fatigue in patients completing the yoga regimen [48]. There was a statistically significant score increase of 7.9 in the yoga group compared to a 0.6 increase in the control group [48]. A significant improvement in fatigue was also reported by Kahraman and colleagues using the Modified Fatigue Impact Scale (MFIS) [50]. Finally, the Rotten Fatigue Severity Scale was used to find a non-significant decrease in fatigue [49].

Discussion

A scoping review was conducted to analyze, collect, and summarize the current findings examining the mental health impacts of yoga for individuals with autoimmune disorders. 11 articles were included in the review covering the autoimmune conditions of MS, RA, and inflammatory bowel disease. The findings indicate that yoga may significantly mitigate mental health concerns associated with chronic autoimmune conditions, including reduction of stress, anxiety, depression, and emotional fatigue, while improving sleep and self-efficacy.

Through the analysis of the methodologies of the articles, a key pattern that was evident within the population of choice was the inclusion of female patients as the predominant population [15,16,18-21,29,31,41,44]. As women have been found to experience autoimmune disorders at a greater rate than men, this was not surprising. Throughout the methodologies of the studies, most yoga programs implemented were not specifically identified or classified, except for two studies that indicated the specific use of Iyengar and Hatha yoga [15,18]. Hatha yoga places emphasis on gentle movements and stretching and has the individual hold postures for a few breaths [55]. Iyengar yoga is similar to Hatha but was adapted as a therapeutic exercise by BKS Iyengar for a variety of health conditions [56]. As yoga practice can be interpreted in multiple formats and styles that overlap with one another, it may be difficult to attribute a therapy regime to one specific yoga method.

In addition to comparing the mental health impacts of yoga use to standard care, four studies implemented an additional group to further examine yoga’s impact on mental health including treadmill training, tele-pilates, aquatic exercise and Baduanjin exercises [15,29,31,41]. Treadmill training consisted of 30-minute sessions three times a week with a target heart rate of 40-75% of the age-predicted maximal heart rate [29]. The aquatic exercise included three one-hour sessions per week that included warming up, endurance activities such as relay races, crossing the pool, strength training, and a cool down [15]. Tele-Pilates consisted of three 60-minute sessions a week including a warmup, the main portion that focused on spine alignment and core engagement, and a cool down [41]. Baduanjin is a traditional Chinese art and science that is used therapeutically for many healthcare needs [31]. The study intervention involved 60-minute daily workouts of eight movements for limbs, body trunk, and eyes [31]. Even when compared with another exercise regime, all four studies noted a greater improvement in the mental health of patients with the use of yoga therapy including reduced anxiety, less fatigue, and decreased depression scores. These findings indicate the potential significance of incorporating non-pharmacological therapies like yoga to mitigate the mental health concerns common among those with autoimmune conditions due to the chronicity of the condition.

It is critical to understand the benefits that yoga can have not only physically but, mentally as well, as those with autoimmune conditions suffer from mental health challenges; however, those are often overlooked with the focus on the physical findings of their condition. It was found that those diagnosed with a variety of autoimmune diseases including RA, psoriasis vulgaris, systemic lupus erythematosus, ulcerative colitis, Graves' disease, MS, Crohn's disease, and type 1 diabetes were at a higher frequency to develop major depressive disorder [57]. Furthermore, the findings may be bimodal as it was found that depressed patients had increased levels of reactivity autoantibodies; conversely, a higher level of autoimmunity related to the development of depression [58-60]. Additionally, autoimmune conditions have been linked with higher rates of anxiety. Joyees et al. found that those with RA, inflammatory bowel disease, systemic lupus erythematosus, and MS had a significantly elevated risk of developing anxiety [61,62]. Specifically, it has been found that RA is an independent risk factor for anxiety [63]. Anxiety and depression have a profound impact on sleep and quality of life. Specifically, in those with autoimmune conditions poor sleep was highly prevalent and connected to depression among patients with RA, MS, psoriatic arthritis, psoriasis, and systemic lupus erythematosus [64-67]. As yoga has previously been shown to impact mental health, this review emphasizes the role yoga can play in this specific patient population.

Chronic inflammatory pathways are the leading mechanism for autoimmune conditions such as RA, MS, and irritable bowel disease. Chronic inflammation is characterized by cytokine overactivity, most notably, cytokine storm. The overactive dysregulated production of inflammatory molecules IL-6, CRP, TNF-α, and interferon-gamma (INF-γ) [15,31]. Chronic increases of IL-6 have been linked to autoimmune conditions and have been functional as a target for the treatment of RA with tocilizumab, an anti-IL-6 receptor antibody [43]. Other markers CRP is a notable regulator of inflammation with its role in cytokine production, complement activation, and nitric oxide production [68]. 

Nugent et al. reported that Hatha yoga markedly decreased inflammatory marker IL-6 in patients with major depressive disorder who were randomly assigned to the Hatha yoga group and attended classes once a week for 10 weeks when compared to the control group receiving health education [44]. This is also supported by findings from Harkess et al., which analyzed levels of IL-6, TNF, and CRP as well as the DNA methylation of each gene in a sample of 28 women reporting psychological stress who underwent eight weeks of yoga intervention. They concluded decreased levels of TNF DNA methylation across the whole gene site [69]. Kaminsky et al. showed similar findings when female patients with RA diagnosis underwent DMARD treatment with Raja Yoga for eight weeks and serum TNF-a, IL-6, CRP, ESR, and IL-17A showed a significant (p < 0.05) decrease as compared to the control group receiving only DMARDs [70].

The unregulated inflammatory response present in autoimmune disease is a target for treatment not only with immunotherapies but also with a holistic approach incorporating Hatha, Iyengar, and Ratha yoga. The reduction of inflammatory markers like IL-6, TNF-α, and CRP through the incorporation of yoga into the treatment regimen of patients with autoimmune conditions, can function to further regulate the immune system and in turn provide physical and mental health benefits.

There were several limitations of the studies analyzed. Firstly, the lack of classification of the type of yoga utilized, if included may have identified specific types of yoga that are more effective than others for patients with autoimmune conditions. There was additionally a wide variability in the type of yoga program utilized and duration of use. Furthermore, the studies did not include follow-ups to allow for long-term analysis of the mental health of this patient following the completion of the yoga program and adherence rate to yoga programs following the conclusion of the studies. Also, the majority of the papers utilized differing scales to compare stress, anxiety, sleep, depression, self-efficacy, and emotional fatigue, leading to difficulty in comparing the effectiveness of yoga between the studies. A gap in the literature requires additional articles to be completed to analyze the impact of yoga outside of MS, IBD, and RA such as in lupus, psoriatic arthritis, Graves' disease, celiac disease, and Hashimoto thyroiditis to truly understand its impact on autoimmune conditions.

To advance the field, future research should standardize yoga protocols, including specific styles, duration, and frequency, to facilitate comparisons across studies, conducting longitudinal studies to evaluate the durability of yoga's mental health benefits and adherence in autoimmune populations. There should be further studies conducted on the long-term use of yoga and adherence to these programs as well as studies conducted in underreprsented autoimmune conditions. The scope of the research includes a broader range of autoimmune diseases and investigates the mechanistic basis of yoga’s anti-inflammatory effects, particularly its influence on cytokine signaling pathways.

## Conclusions

Living with a chronic illness can be both physically and mentally debilitating. Yoga offers a unique and holistic solution to mental health in patients with autoimmune disorders. Although many reviews have discussed the impacts of yoga on physical health, they fail to recognize the mental health component. The results of this review suggest that the implementation of yoga should be considered in medical practice as a viable alternative in place of or adjunct to pharmaceutical options when treating patients with autoimmune disorders. The review indicates that yoga can benefit patients by decreasing stress, anxiety, depression, self-efficacy, and emotional fatigue. While medications can have an important role in controlling the progression of autoimmune disorders, some individuals may not tolerate the medications or their side effects. Yoga is a generally safe and minimal-risk practice that can easily be incorporated into a patient's therapeutic regimen. Medical practitioners are encouraged to introduce yoga therapy to their patients and support its integration into the patient’s daily routines, allowing patients to feel empowered in the management of their own health. Long-term studies are required to further understand the effectiveness and feasibility of introducing yoga programs into treatment regimes for patients as well as further understanding its use amongst all autoimmune conditions in order to treat patients with evidence-based therapeutic approaches. Physicians should explore and promote the options of yoga as a useful tool in managing symptoms of autoimmune disorders to incorporate a more holistic approach, slowly moving away from only pharmaceutical therapeutics. 
